# Major surgical approaches to the posterior third ventricular region: A pictorial review

**DOI:** 10.4103/1817-1745.76093

**Published:** 2010

**Authors:** Sanjay Behari, Pallav Garg, Sushila Jaiswal, Anup Nair, Ram Naval, Awadhesh K. Jaiswal

**Affiliations:** Department of Neurosurgery, Sanjay Gandhi Postgraduate Institute of Medical Sciences, Lucknow, India; 1 Department of Pathology, Sanjay Gandhi Postgraduate Institute of Medical Sciences, Lucknow, India

The lesions of the pineal gland, posterior third ventricle and the dorsal midbrain have been approached by many approaches. All of them require precision and knowledge of the anatomy of this region. Horsley was the first to attempt surgery for these lesions. The credit for the first successful excision of tumors of this region goes to Krause in 1913 who utilized the infratentorial supracerebellar approach,[1] an approach revived by Stein in 1971. The alternative approaches to this region are Jamieson’s[2] and Poppen’s[3] occipital transtentorial approach, Van Wagenen’s posterior transventricular approach[4] and Dandy’s posterior transcallosal approach.[5–7]

## Infratentorial Supracerebellar Approach

**Figure 1:**
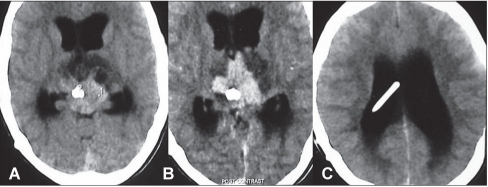
Patient 1: (A-C): This 8-year old boy presented with raised pressure, bilateral VIth nerve and upward gaze palsy of 8-months duration. His contrast enhanced CT scan showed a uniformly enhancing, infiltrative, posterior third ventricular lesion reaching the anterior third ventricle nearly until the foramen of Monro and causing hydrocephalus. The right ventriculoperitoneal shunt initially placed at another center got blocked and required revision.

**Figure 2:**
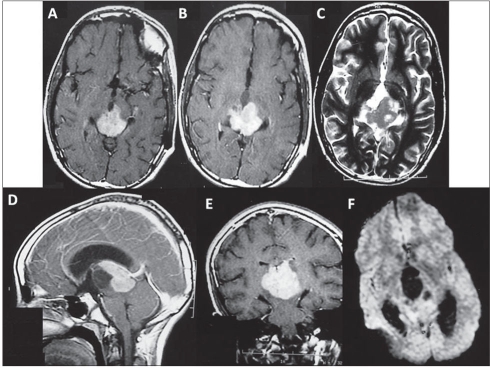
Patient 1: A and B: Contrast enhanced axial T1-weighted MRI following the ventriculoperitoneal shunt revision showed the uniformly enhancing infiltrative lesion occupying the posterior third ventricular region and the quadrigeminal cistern. C: T2-weighted axial image showing the lesion to be heterogeneously iso- to hyperintense. D: Sagittal contrast enhanced T1 image showing the lesion occupying the third ventricle along the internal cerebral vein almost reaching upto the foramen of Monro. E: The coronal-enhanced T1 image showing the vertical extent of the lesion from the foramen of Monro to the midbrain. F: The diffusion-weighted image showing restriction of diffusion within the lesion.

This is the commonly used approach for lesions of pineal gland, dorsal midbrain and superior vermis [Figures 1–5].

**Figure 3:**
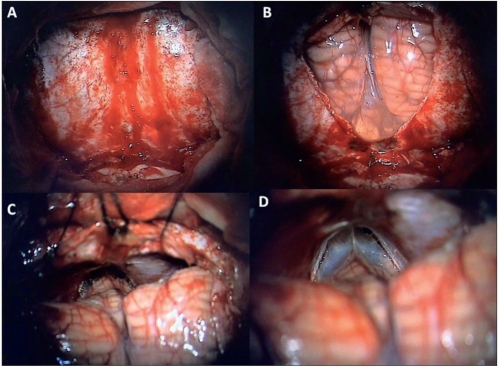
Patient 1: Infratentorial, supracerebellar approach was adopted in sitting position. A: A midline linear incision through the skin and ligamentum nuchae exposed the occipital bone from the external occipital protuberance to the foramen magnum. The craniectomy revealed the exposed rim of the transverse and occipital sinus, and the dura covering bilateral cerebellar hemispheres and foramen magnum. B: The dura is opened with a “Y” shaped incision coagulating the occipital sinus and the annular sinus (the latter at the foramen magnum) and refl ected superiorly along the transverse sinus. C: The anastomotic veins between the superior surface of cerebellar hemispheres and the tentorium are coagulated allowing the cerebellum to fall with gravity away from the tentorium and creating the space for the surgical approach. D: The arachnoid covering the tumor in the posterior third ventricular region and the precentral cerebellar vein in the midline are seen due to gravity-assisted fall of the cerebellum.

**Figure 3:**
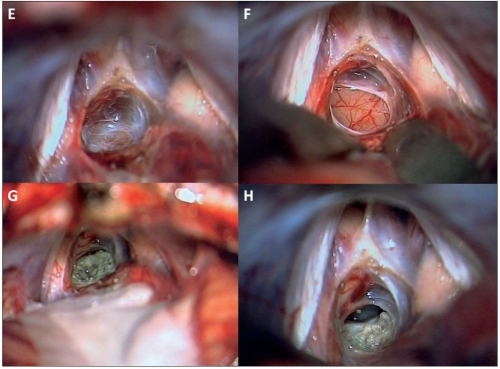
Patient 1: E: The precentral cerebellar vein is coagulated and divided. F: The arachnoid covering the tumor is removed exposing the tumor surface. G: The tumor is gently coagulated and removed in a piecemeal manner. H: The opening of the third ventricle following tumor removal drains CSF.

**Figure 4A:**
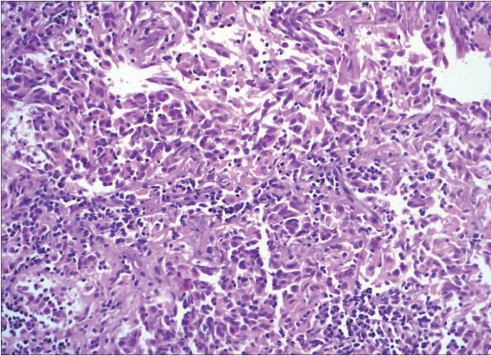
Patient 1: Photomicrograph showing round to polygonal tumor cells disposed in groups, displaying conspicuous nucleoli at places and variable amount of pale to amphophilic cytoplasm. The groups of tumor cells are separated by fibrous septa infiltrated by small mature lymphocytes (H and E, ×400)

**Figure 4B:**
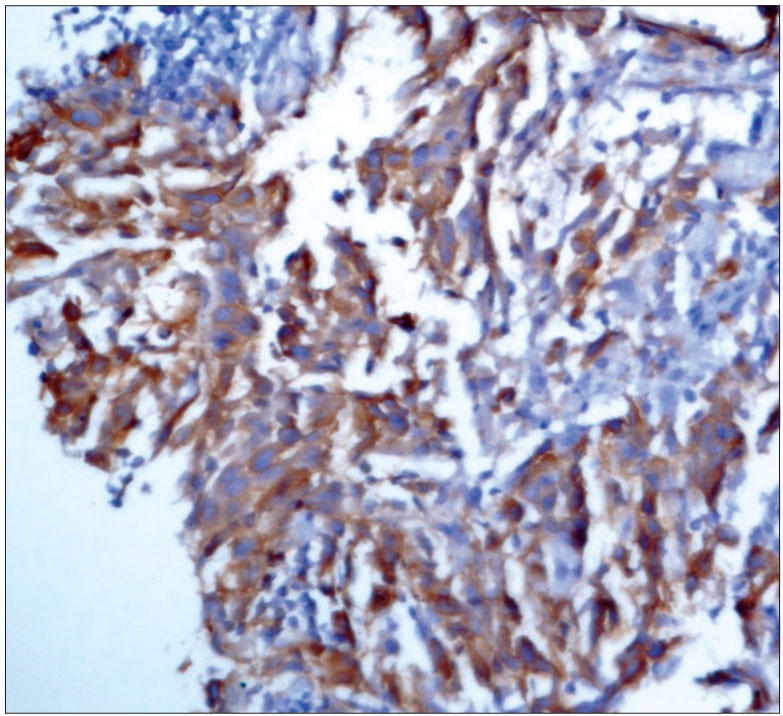
Patient 1: Photomicrograph showing tumor cell positive for CD 117 (Immunohistochemical stain; ×400)

**Figure 4C:**
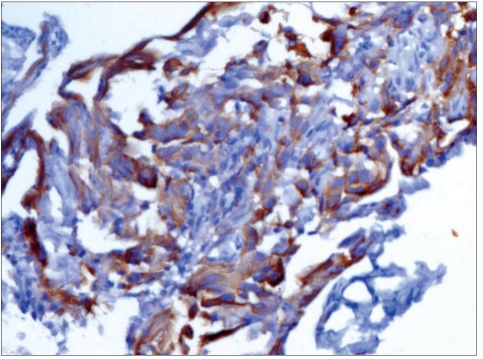
Patient 1: Photomicrograph showing tumor cell positive for placenta-like alkaline phosphatase (PLAP) (Immunohistochemical stain; 400×) The histopathology and immunohistochemistry confirmed the presence of a GERMINOMA.

**Figure 5:**
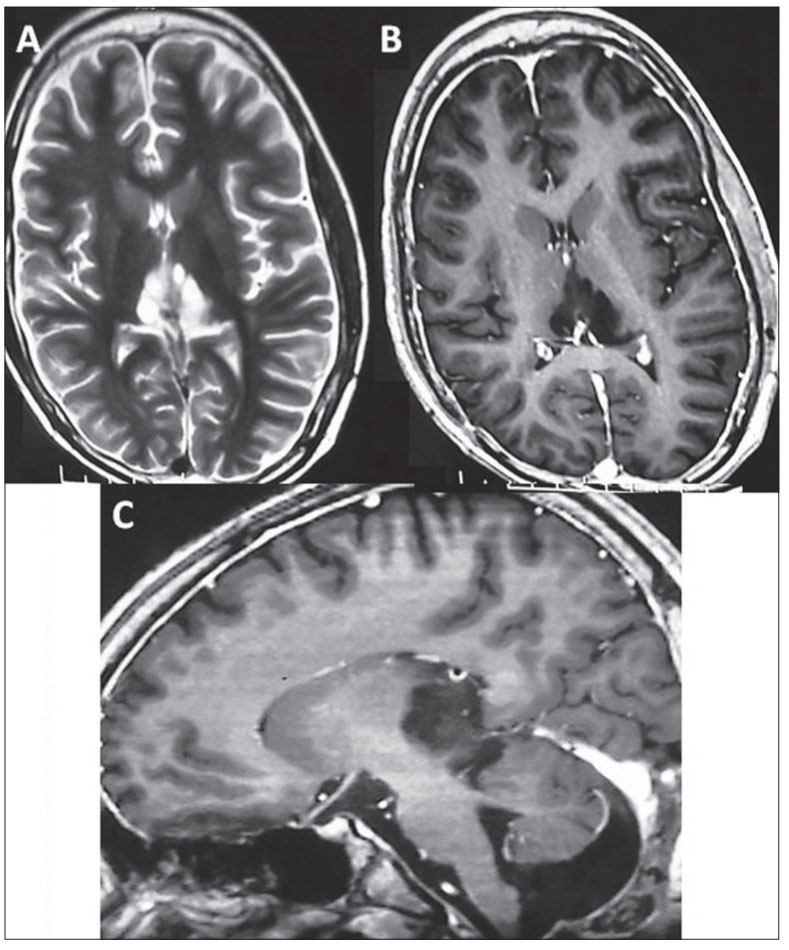
A: Patient 1: Axial T2; B: axial contrast T1; and, C: Sagittal contrast T1-weighted images after surgery and radiotherapy showing a small nonenhancing component of the residual lesion with no hydrocephalus.

### Advantage

The midline trajectory of the approach to the tumor avoids injury to the deep venous channels (the internal cerebral veins draining into the vein of Galen that in turn drains into the straight sinus; and, the basal vein of Rosenthal that traverses the ambient cistern and drains into the vein of Galen) that usually are located superior to the lesion. The approach is toward the center of the tumor from where it may be extended eccentrically. There is a good exposure with minimal neural damage. The sitting position offers a good exposure with gravity-assisted drainage of blood and cerebrospinal fluid (CSF).

### Disadvantage

Lesions with a significant component extending laterally upto the trigone of the lateral ventricle or lesions involving the corpus callosum are difficult to completely remove by this approach and require supratentorial approaches. The sitting position in which this surgery is usually performed increases the risk of air embolism, tension pneumocephalus and hyperflexion injury leading to quadriplegia.[7]

### Position

The sitting position is the preferred position for this approach although a three-fourth prone or lateral decubitus position has also been used. The latter positions are used in the pediatric age group particularly under 2 years of age. The head in the sitting position must be adequately flexed to align the tentorium in the horizontal position for an adequate exposure of the supravermian infratentorial space.

### Incision

A midline vertical incision extending from the C3 spinous process to 2-3 cm above the external occipital protuberance is utilized. The muscles of the suboccipital and posterior cervical region are retracted after dividing the avascular ligamentum nuchae in the midline. The pericranium covering the occipital bone is dissected off the bone. The suboccipital craniectomy or craniotomy exposes the rim of the torcula and the transverse sinus superiorly and reaches inferiorly until the foramen magum.

### Operative Steps

The dura is opened in a “V” or a “Y” shaped manner with the base toward the transverse sinus. In case the surgical field of view is not adequate, the bridging veins from the dorsal cerebellar surface to the tentorium may be sacrificed to permit the gravity-dependent descent of the cerebellum. Gentle retraction with self-retaining retractors aids in depressing the cerebellum for establishment of the operative corridor between its superior vermian surface and the inferior surface of tentorium.

The arachnoid of the quadrigeminal cistern is thickened and opaque. The midline precentral cerebellar vein traversing vertically downward in the midline just anterior to the thickened arachnoid may either be retracted or coagulated. This permits a trajectory toward the quadrigeminal cistern, velum interpositum, collicular plates and the third ventricle. Once the tumor is encountered, internal decompression is done followed by careful dissection from the surrounding structures including the deep venous system superiorly and the brain-stem, collicular plates and the thalamus anteriorly. Following tumor decompression, the third ventricular cavity and the lining ependyma is well-visualized right until the foramen of Monro. In case of dense adhesions of the tumor capsule to the surrounding vital structures, it is preferable to leave parts of the capsule than risk retraction injury by persisting with its complete removal.

In patients with significant hydrocephalus, a preoperative CSF diverting procedure may be employed before the definitive surgery.

### Complications

Postoperative complications include CSF leak and acute or delayed hemorrhage.[6] This may be due to bleeding within the residual tumor. Alternatively, a point of bleeding may be missed during hemostasis due to gravity-dependent collapse of the bridging veins in sitting position. The rent may open up in the postoperative period when the patient is made supine and his blood pressure increases on reversal from anesthesia. Hemorrhagic venous infarction may also occur due to coagulation of a bridging vein.

## Occipital transtentorial approach

**Figure 6:**
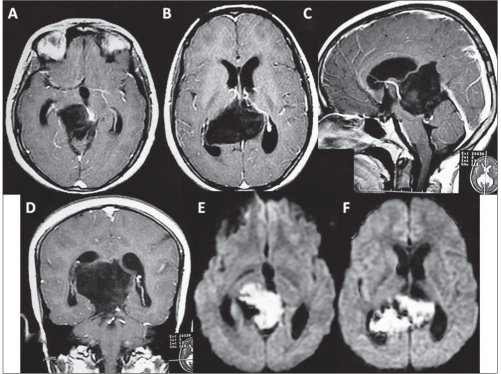
Patient 2: A, B: This 16-year-old girl presented with symptoms of raised intracranial pressure, Perinaud’s syndrome and gait ataxia. Her axial contrast T1-weighted images showed the nonenhancing irregular lesion in the posterior third ventricular region and the ambient and quadrigeminal cisterns. There is mild hydrocephalus with dilated anterior third ventricle and bilateral lateral ventricles. C: Sagittal, and D: Coronal contrast T1 images showing the vertical extent of the lesion along the brain stem and its extension into the supratentorial compartment after occupying the tentorial incisural space. The superior vermis of the cerebellum appears flattened by the lesion. E, F: Diffusion-weighted axial images showing the restriction of diffusion in the lesion.

This approach [Figures 6–9] provides adequate exposure of both the superior and inferior surfaces of the tentorial notch and hence is excellent for tumors straddling the tentorial notch. This approach is also useful for tumors situated above the confluence of the deep venous system and for tumors extending laterally into the trigone of the lateral ventricle.[7]

**Figure 7:**
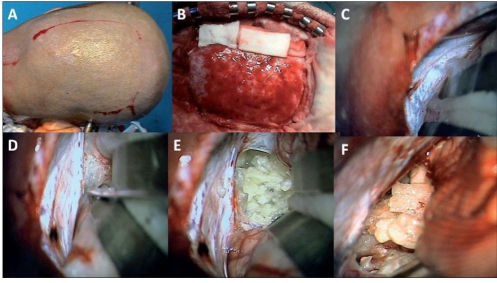
Patient 2: A: Right occipital craniotomy, posterior interhemispheric, transtentorial approach adopted in prone position with the table tilted about 20-30° toward the side of approach to permit a gravity-dependent spontaneous retraction of the right parieto-occipital lobe facilitating the approach through the posterior interhemispheric corridor that is usually devoid of bridging veins. B: The dural exposure after the craniotomy that extends until the rim of the torcula and transverse sinus inferiorly and the posterior part of the superior sagittal sinus medially. The dura is cut in a square shape and refl ected medially based upon the superior sagittal sinus. C: Gentle lateral retraction of the right parieto-occipital lobe exposes the falx cerebri and its junction with the tentorium that encloses the straight sinus within its leaves at the junctional area. D: The tentorium is traced until its incisura parallel to the straight sinus. The epidermoid tumor is visible within its arachnoid covering at the incisura. E: The tentorial surface is coagulated and divided parallel and slightly away from the straight sinus and the arachnoidal covering of the epidermoid removed exposing the tumor. F: The tumor is removed in a piecemeal manner with the help of microdissectors, gentle irrigation and suction.

**Figure 7:**
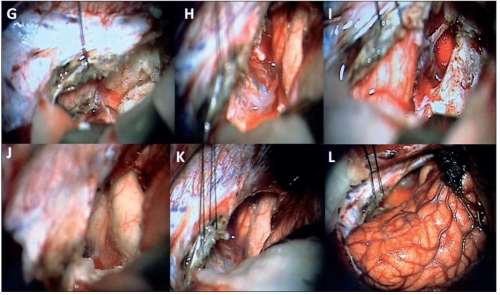
Patient 2: G: Following tumor removal, the superior surface of cerebellum and brain stem are exposed. The divided tentorium is being retracted with stay sutures providing an adequate corridor. There is a thin capsule of the epidermoid still lining the brain stem, and H: the superior surface of the cerebellum. I: The vein of Galen and basal vein of Rosenthal, and J: the posterior thalamus, collicular plate and the quadrigeminal cistern are visible following tumor removal. K: The cerebellum, brain stem, and, L: the lax brain after the procedure. A bridging vein at the anterior end of the corridor is protected with surgicel.

**Figure 8:**
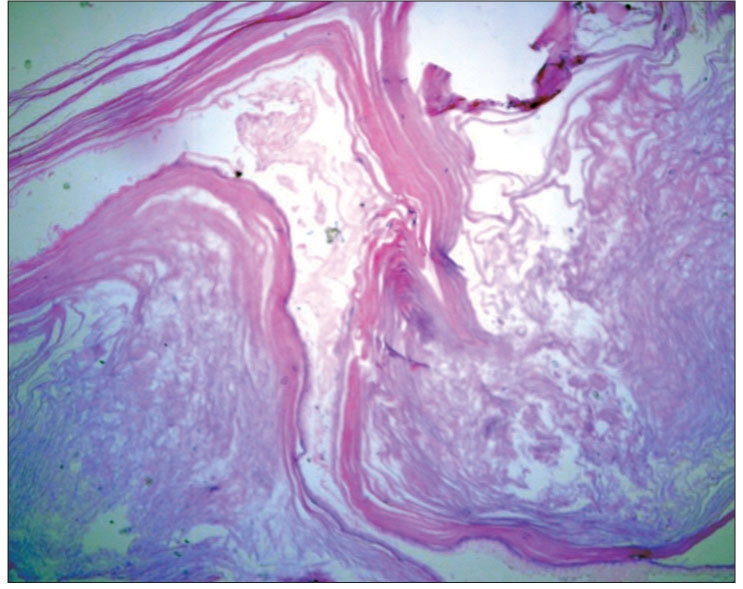
Patient 2: Photomicrograph showing a cyst lined by thinned out stratified squamous epithelium filled with keratin material suggestive of epidermoid (H and E, ×400)

**Figure 9:**
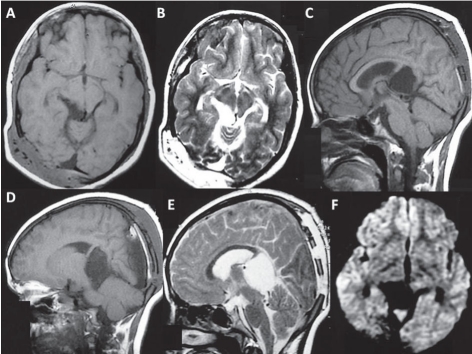
Patient 2: A: axial T1; B: axial T2; C, D: sagittal T1; E: sagittal T2; and F: diffusion-weighted axial MR images showing the postoperative tumor cavity after total excision of the lesion.

### Position

The patient is placed in prone position or lounging position. In prone position, the table is slightly tilted to the ipsilateral side to facilitate gravity-dependent retraction of the occipital lobe from the falx cerebri.

### Incision

A square or triangular scalp flap with the longitudinal limb being in the midline and extending from just below the external occipital protuberance to 6-8 cm superior to this point and curving laterally and downwards is used. Preferably the approach is carried out from the nondominant side.

### Operative steps

The craniotomy is fashioned to expose the rim of the transverse sinus inferiorly, the superior sagittal sinus medially and it extends laterally upto 5-6 cm. The dural opening may be as two triangular flaps with the base toward the transverse and sagittal sinuses, respectively, or as a square flap medially toward the superior sagittal sinus. In case hydrocephalus is coexistent, the posterior horn of the lateral ventricle may be tapped to facilitate CSF drainage and brain retraction.

The occipital lobe is gently retracted laterally. This maneuver is facilitated by the fact that bridging veins between the cerebellar surface and the superior sagittal sinus are few and small and may usually be divided with very little risk of venous infarction of the occipital lobe. The falx cerebri, the tentorium and the dural covering of the straight sinus at the junctional region of these dural folds are visualized from the region of the torcula to the tentorial incisura. The tentorium is coagulated parallel and lateral to the straight sinus (avoiding any venous lakes in close proximity to the straight sinus) and divided until the incisural edge thereby bringing the superior cerebellar surface in view. The divided leaflets of the tentorium are reflected using stay sutures. The opaque and tough arachnoid over the deep veins is left intact if the tumor is below their level. Working in the arachnoidal plane below the major veins, lesion of the posterior third ventricular regions are exposed. The tumor removal is then carried out in a piecemeal manner. Following tumor removal, the brain stem, the collicular plate and the posterior thalamic regions are well-visualized.

### Advantage

Adequate exposure both above and below the tentorial notch is available. Lesions reaching upto the trigone and corpus callosum are accessible. This approach is specially preferred in cases where the deep venous system is dorsally displaced as occurs in tentorial meningiomas.

### Disadvantage

In tumors extending eccentrically to the contralateral side, complete removal is difficult. Care must be taken to avoid damage to the occipital lobe (that may precipitate homonymous hemianopia) and the splenium of corpus callosum (that may result in posterior disconnection syndrome).

### Neurological Complications

Regardless of the approach, Perinaud’s syndrome with upward gaze palsy, pupillary and accommodation abnormalities with partial or complete oculomotor nerve palsy, trochlear nerve injury with diplopia or even alteration of sensorium due to brain stem injury may result.

## Other Approaches

The posterior interhemispheric transcallosal approach utilizes a parietal, interhemispheric approach to gain access to the lesions situated above the deep venous system and involving the posterior corpus callosum. The anterior transcallosal, transventricular approach is useful when the tumor occupies lateral ventricles and posterior third ventricles. It utilizes the conventional anterior interhemispheric approach to gain access into the lateral ventricle by creating a small opening in the corpus callosum. The access from the lateral to the third ventricle is gained by a subchoroidal approach or an approach medial to the choroid plexus by dividing the thin velum interpositum that is encountered and, by following a trajecteory that is directed posteriorly toward the pineal region infero-lateral to the internal cerebral veins traversing the roof of the third ventricle. Finally, lateral paramedian infratentorial approach in park-bench position traverses between the superior surface of cerebellar hemisphere and the lateral tentorium. The trajectory is directed superomedially toward the tentorial incisura. The bridging veins traversing this corridor may be divided to gain additional space.[7–9] Stereotactic or endoscopic biopsy may also be utilized to identify lesions that have an excellent response to radio- and chemotherapy, thus avoiding the need for a major surgery.

In conclusion, posterior third ventricular tumors are frequently encountered in children. Their surgical excision is technically demanding. The results, however, are gratifying as the tumors are often soft-suckable or well-marginated permitting total removal with minimal morbidity and mortality. The residual lesions, if any, may often have a good therapeutic response to radiotherapy and chemotherapy.
